# Variants in Adjacent Oxytocin/Vasopressin Gene Region and Associations with ASD Diagnosis and Other Autism Related Endophenotypes

**DOI:** 10.3389/fnins.2016.00195

**Published:** 2016-05-12

**Authors:** Sunday M. Francis, Emily Kistner-Griffin, Zhongyu Yan, Stephen Guter, Edwin H. Cook, Suma Jacob

**Affiliations:** ^1^Department of Psychiatry, University of MinnesotaMinneapolis, MN, USA; ^2^Biostatistics Shared Resource, Hollings Cancer Center, Medical University of South CarolinaCharleston, SC, USA; ^3^Dow AgroSciences LLCIndianapolis, IN, USA; ^4^Department of Psychiatry, Institute of Juvenile Research, University of Illinois at ChicagoChicago, IL, USA

**Keywords:** oxytocin, vasopressin, ASD, genetics, polymorphisms, biomarkers, phenotypes, IQ

## Abstract

**Background:** There has been increasing interest in oxytocin (peptide: OT, gene: *OXT*) as a treatment pathway for neurodevelopmental disorders such as Autism Spectrum Disorder (ASD). Neurodevelopmental disorders affect functional, social, and intellectual abilities. With advances in molecular biology, research has connected multiple gene regions to the clinical presentation of ASD. Studies have also shown that the neuropeptide hormones OT and arginine vasopressin (AVP) influence mammalian social and territorial behaviors and may have treatment potential for neurodevelopmental disorders. Published data examining molecular and phenotypic variation in ASD, such as cognitive abilities, are limited. Since most studies have focused on the receptors in the OT-AVP system, we investigated genetic variation within peptide genes for association with phenotypic ASD features that help identify subgroups within the spectrum.

**Methods:** In this study, TDT analysis was carried out utilizing FBAT in 207 probands (156 trios) and a European Ancestry (EA) subsample (108 trios).The evolutionarily related and adjacent genes of *OXT* and *AVP* were studied for associations between the tagged single nucleotide polymorphisms and ASD diagnosis, social abilities, restrictive and repetitive behaviors, and IQ for cognitive abilities. Additionally, relationships with whole blood serotonin (WB5HT) were explored because of the developmental relationships connecting plasma levels of OT and WB5HT within ASD.

**Results:** Results indicate significant association between OXT rs6084258 (*p* = 0.001) and ASD. Associations with several endophenotypes were also noted: OXT rs6133010 was associated with IQ (full scale IQ, *p* = 0.008; nonverbal IQ, *p* = 0.010, verbal IQ, *p* = 0.006); and OXT rs4813625 and OXT rs877172 were associated with WB5HT levels (EA, *p* = 0.027 and *p* = 0.033, respectively). Additionally, we measured plasma OT (pOT) levels in a subsample (*N* = 54). Results show the three polymorphisms, OXT rs6084258, OXT rs11697250, and OXT rs877172, have significant association with pOT (EA, *p* = 0.011, *p* = 0.010, and *p* = 0.002, respectively).

**Conclusions:** These findings suggest that SNPs near *OXT* and *AVP* are associated with diagnosis of ASD, social behaviors, restricted and repetitive behaviors, IQ, pOT, and WB5HT. Future studies need to replicate these findings and examine gene-interactions in other neurodevelopmental disorders. Mechanisms of action may influence early social and cognitive development that may or may not be limited to ASD diagnosis.

## Introduction

The DSM-5 (American Psychiatric Association, 2013) describes autism spectrum disorder (ASD) as a group of disorders characterized by persistent deficits in social communication and interaction across multiple contexts, and the presence of restricted, repetitive patterns of behaviors (RRBs). It places ASD within a larger category of neurodevelopmental disorders that also includes intellectual disabilities, communication disorders, attention-deficit/hyperactivity disorder, specific learning or motor disorders, and others that continue on to adulthood. ASD is heritable and highly heterogeneous with a complex inheritance process (Bailey et al., 1995). Molecular technology has allowed us to identify numerous contributions to ASD and their related developmental features. Recent changes in DSM-5 reflect a shift from discrete categorization toward broader spectrum, multidimensional characterization of clinical disorders including neurodevelopmental disorders.

Neurohypophysial hormones such as oxytocin (peptide: OT, gene: *OXT*) and vasopressin (peptide: AVP, gene: *AVP*) have been studied increasingly over the last decade, especially in ASD. Located on chromosome 20, *OXT* and *AVP* are closely linked and are positioned in opposite transcriptional orientations approximately 10 kilobases (kb) apart. Recently, there was a study reporting a replication of linkage in this 20p13 chromosome region area for ASD risk genes (Weiss et al., 2009; Werling et al., 2014). OT (and *OXT*) and AVP have been evolutionarily conserved in both structure and function across a diverse range of species, and are involved with social cognition and repetitive behaviors (for review see Jacob et al., 2011). For example, centrally administered OT (Carter et al., 1995) facilitated partner-preference formation, whereas blocking the OT receptor (OTR) inhibited the behavior in monogamous prairie voles. Alternatively, AVP acting at the vasopressin-1A receptor can induce repetitive aggressive behaviors; this effect of AVP was blocked by the simultaneous use of a serotonin (5HT_1*A*_) agonist (for review of animal studies see Carter and Jacob, 2012).

Receptor genes for OT and AVP have been researched to examine relationships with many aspects of social behavior. Parker et al. (2014) found the OT receptor gene (*OXTR*) SNP rs2254298 to be associated with social impairment in both an ASD and neurotypical sample. Recent studies have revealed a role for the neuropeptide hormones in social withdrawal disorders and as a potential treatment pathway for ASD and schizophrenia (Pedersen et al., 1994; Kendrick et al., 1997; Wang and Young, 1997; Ferguson et al., 2000, 2001; Young, 2001; Carter, 2007; Souza et al., 2010a,b; Teltsh et al., 2012; Anagnostou et al., 2014). In ASD, there have been linkage and association studies for OT and AVP pathway genes (for review see Francis et al., 2014b) including the disruption of *CD38*, which is involved in OT secretion (Lerer et al., 2010; Munesue et al., 2010; Ceroni et al., 2014). The relationship between OT and ASD has also been strengthened by research examining OT levels in plasma. An early study showing that plasma OT (pOT) concentrations were lower in children with ASD (Modahl et al., 1998), led to an OT deficit model that has been tested in subsequent studies with ASD populations with varying results. Several groups have reported low pOT in ASD (Al-Ayadhi, 2005; Andari et al., 2010), and others no difference (Miller et al., 2013) or high pOT (Jansen et al., 2006) in ASD. Researchers have also shown that lower pOT is associated with decreased social functioning within neurotypical cohorts as well (Clark et al., 2013; Feldman et al., 2014).

In individuals with ASD, we previously observed a correlation between whole-blood serotonin and plasma OT levels (Hammock et al., 2012). OT and serotonin (5-HT) were negatively correlated with each other (*p* < 0.05) and this relationship was most prominent in children less than 11 years old. Of the many biomarkers studied in ASD, the oxytocinergic and serotonergic systems have numerous connections. In animal as well as human studies, both 5-HT and OT were able to modulate the release of each other depending on brain location (Sawchenko et al., 1983; Emiliano et al., 2007; Hammock et al., 2012). In 2009, Yoshida et al. demonstrated modulation of 5-HT in the raphe nuclei by the OT system (Yoshida et al., 2009). The raphe nuclei are a core area of 5-HT synthesis. Recently, in 2014, an imaging study utilized PET and a marked selective 5-HT_1A_ antagonist to map the 5-HT_1A_ system by administering OT and placebo to healthy males (Mottolese et al., 2014). They observed a decrease in extracellular 5-HT in the dorsal raphe nucleus, amygdala-hippocampus-parahippocampus complex, insula and orbitofrontal cortex (mid/ventral) noting that OT modulated the serotoninergic system. It is hypothesized that the amygdala-hippocampus complex may be where the interactions of OT and 5-HT regulate responses to stress and anxiety. Given our previous research regarding changes in whole-blood serotonin (WB5HT) and plasma OT levels with puberty and age, and other studies examining the interactions between the oxytocinergic and serotonergic systems, further research is required to understand the possible roles and interactions of these two systems as they relate to symptom domains in early and later development.

As with studying the role of OT and AVP in social behavior, *OXTR* was an initial point for researchers to begin exploring the role of OT and AVP in ASD (Lerer et al., 2008; Tansey et al., 2010; Wermter et al., 2010; Lakatosova et al., 2013). Two recent meta-analyses have also reported associations between *OXTR* and ASD (LoParo and Waldman, 2015; Kranz et al., 2016). LoParo and colleagues analyzed data from eight studies and 11 independent samples, finding associations between ASD and the *OXTR* single nucleotide polymorphisms (SNPs)—rs7632287, rs2268491, and rs2254298. Our lab previously reported an association between rs2254298 and ASD in a Caucasian sample (Jacob et al., 2007). Kranz et al. (2016) analyzed two independent German samples and 10 additional studies, finding an association between ASD and rs237889. While we have focused on OT, AVP, and their receptors, multiple genes and biomarkers are involved with ASD risk. Researchers are finding ways to study pathway interactions that contribute to specific phenotypes. Intermediate phenotypes, also called endophenotypes, have distinct heritable components of the overall disorder and reduce heterogeneity (Gottesman and Gould, 2003). ASD researchers often parse clinical heterogeneity by measuring levels of social communication and restricted/repetitive behaviors (RRB) subphenotypes in order to investigate underlying biological systems more directly (Abrahams and Geschwind, 2008; Levin-Decanini et al., 2013). These two quantitative traits have phenotypic variability and can be attributed to many genes. Although most of this work has focused on *OXTR*, two *OXT* SNPs (rs2740210 and rs4813627) were associated with maternal care giving measures (e.g., motherese vocalizations during mother-infant interactions; Mileva-Seitz et al., 2013). Given the roles of OT and AVP in social behavior, pathway genes have been studied in parental behaviors, pair bonding, social motivation, social memory, social preference, social competence, empathy, social cognition or theory of mind, social anxiety, and stress management with performance pressures (for review see Meyer-Lindenberg et al., 2011; Feldman et al., 2015).

Fewer human studies have examined RRB as a quantitative phenotype with OT pathways genes, although recently a study of *OXT* showed effect for repetitive as well as social subphenotypes in the 3′UTR of the OT gene (Harrison et al., 2015). Animal studies have also suggested that cognitive rigidity may be a RRB-like subphenotype related to *OXT* and ASD (Sala et al., 2011). Restricted/repetitive behaviors have a range of features including compulsions, ritualistic/sameness, restricted, stereotyped, and self-injurious behaviors that may be influenced by OT or AVP pathways (for review see Francis et al., 2014a). Although, most research has focused on the receptor genes, a few studies have suggested that SNPs in *OXT* and *AVP* are associated with RRBs. In a study of ASD and hormonal genes, OXT rs2740204 was associated with stereotyped behaviors but not overall diagnosis (Yrigollen et al., 2008). Additionally, in 2009, Ebstein and colleagues genotyped 170 subjects with ASD testing both individual SNPs and haplotypes in *OXT*. Nominal associations were observed between ASD and OXT rs6133010, as well as two-, three-, and four-locus haplotypes. In a Swedish twin study, the researchers observed an association between OXT rs2770378 and autism-like traits including language impairment and restricted behaviors in females but not males with ASD (Hovey et al., 2014).

There are also limited data on molecular associations with discrepant cognitive abilities, another important ASD endophenotype (Lerer et al., 2008; Chapman et al., 2011). Intellectual disability as defined by the DSM-5 involves impairments of general mental abilities that impact adaptive functioning in everyday tasks across three domains (conceptual, social and practical). The removal of IQ scores (IQ ≤ 70) from the diagnostic criteria of intellectual disability, but still including them in the text description, emphasizes that an important aspect of a person's intellect is a measure of his/her overall general mental ability centered around social behavior, which is the core deficit in ASD. Increased discrepancies between performance and verbal IQ (cognitive profile) have been observed in ASD individuals and have correlated with core components of the ASD phenotype. A review of 23 studies considering the cognitive profile of individuals with autism found that verbal IQ (VIQ) is generally lower than performance IQ (Lincoln et al., 1988), a pattern found in a recent study of over 450 preschoolers with autism (Munson et al., 2008). This decreased VIQ relative to performance IQ could be interpreted as an indirect measure of social communication impairment. In ASD research conducted in 2008, Lerer and colleagues found a nominal yet significant association with IQ and two *OXTR* SNPs (rs4686301 and rs1042778). Next they performed haplotype analysis ranging from two to eight markers along a sliding window. IQ was found to be significantly associated with several haplotypes ranging across all haplotype lengths. Also when IQ was entered as a covariate, significant associations were observed with two *OXTR* SNPs (rs2268494 and rs1042778) and ASD.

Given the previously published associations between the OT-AVP system and behaviors and phenotypes in ASD, we intend to further elicit associations between tagged SNPs and haplotypes, and social behavior, ASD diagnosis, social phenotypes, and IQ. Currently, there is very little functional data about OT and AVP pathway gene SNPs and how they relate to functional changes in human tissues, especially within the brain. There is limited literature on receptor distribution, cerebrospinal fluid, and neuroglial vesicular transport of OT and AVP neuropeptides and the range of affected neurocircuits in humans. Current studies are being done in human brain pathology tissues using receptor ligands that have been successful in non-human primates. We selected expression quantitative trait loci (eQTL) analysis to investigate expression as a first step in looking at functional expression changes. We also explored these relationships in association with two biomarkers: pOT and whole blood 5-HT (WB5HT). The 5-HT and OT systems interact in the brain, both during development and in adulthood. The separate lines of evidence for blood 5-HT and OT measures as biomarkers in ASD, coupled with the evidence for intersection between the corresponding systems in the brain, led us to further examine this relationship.

## Materials and methods

### Participants

With the approval of the University of Illinois at Chicago (UIC) Institutional Review Board (IRB#: 2007-0239; Title: Interdisciplinary Studies of Insistence on Sameness (IS) in Autism Spectrum Disorders (ASD)), subjects were recruited through the Developmental Disorders Clinic of the UIC Institute for Juvenile Research, referral from providers of autism services, a website providing information about the study, and parent advocacy organizations. Prior to the first session, all participants were provided a description of the study to obtain informed consent from adults able to consent for themselves, and parents or guardians of minors and individuals unable to consent. All subjects had a medical history and physical examination performed by a pediatric neurologist or child psychiatrist, and a psychiatric evaluation by a child psychiatrist experienced in ASD. Probands were assessed utilizing the Autism Diagnostic Observation Schedule (ADOS; Lord et al., 2000) and Autism Diagnostic Interview—Revised (ADI-R; Lord et al., 1994) and were required to meet ASD or autism criteria on the ADOS and autism criteria or ASD criteria on the ADI-R; the ASD diagnosis was confirmed by clinical consensus of a clinical psychologist and child psychiatrist experienced in ASD diagnosis in accordance with the DSM-IV-TR ASD classification (including autism, Asperger disorder, or pervasive developmental disorder-not otherwise specified (PDD-NOS)). One hundred fifty-six probands completed genotyping for both mothers and fathers for the trio design. We had data from an additional 51 probands with only one genotyped parent. Some biological measures like WB5HT are influenced by ethnicity therefore we repeated analyses on a restricted sample (108 complete trios) based on their European ancestry (EA). Race and ethnicity information was obtained during an initial screening by parent or self-report. Table 1 describes demographic data for the entire sample and the EA subsample. The data supporting the conclusions in this article are available through the NIH sponsored database, National Database for Autism Research (NDAR; https://ndar.nih.gov/study.html?id=404).

**Table 1:** **Demographic description of the entire genotyped sample and the genotyped European Ancestry (EA) subsample**.

	**All Probands**	**EA Probands**
Number Genotyped	207	108
Average Age (years)	9.88 (±5.48 SD)	9.82 (±5.99 SD)
Sex	170 (82.1% male)	91 (84.3% male)
	37 (17.9% female)	17 (15.7% female)
**IQ MEASUREMENTS**		
Full-Scale IQ	79.9 (*N* = 193; ±24.0 SD)	83.9 (*N* = 103; ±24.0 SD)
Verbal IQ	77.5 (*N* = 196; ±26.0 SD)	83.6 (*N* = 102; ±26.2 SD)
Non-verbal IQ	82.1 (*N* = 204; ±23.9 SD)	85.5 (*N* = 108; ±23.1 SD)

The EA subsample utilized in the biomarker analysis was pulled from the original, full dataset.

The IQ and social subphenotypes of the participants were measured utilizing assessment tools appropriate for the subjects' ages and abilities. The Differential Ability Scales, Second Edition (DAS-II; Elliot, 2006) was the primary and preferred assessment for preschool through adolescent age participants. Mullen Scales of Early Learning (MSEL; Mullen, 1995) was administered to individuals with low cognitive abilities, and the Wechsler Abbreviated Scale of Intelligence (*WASI;* Wechsler, 1999) for higher functioning subjects ≥18 years of age. A standard IQ score was calculated based on the norms of the various tests, measured mental age(s), and chronological age. We assessed full-scale IQ (FSIQ) in our study, and then subdivided FSIQ into verbal (VIQ) and non-verbal IQ (NVIQ), which we examined separately.

Two instruments administered to assess social abilities were the Aberrant Behavior Checklist Community Version (ABC-CV; Aman et al., 1985) and the Social Responsiveness Scale (SRS; Constantino and Todd, 2000). ABC-CV, a five-factor, 53 item assessment was completed by the parent or guardian with direct knowledge of the subject. This instrument, approved for individuals between 6 and 54 years old, assessed symptoms of irritability and agitation, social withdrawal (or lethargy), stereotypic behavior, hyperactivity and non-compliance, and inappropriate speech. To measure the severity of autism symptoms in social settings, we utilized the SRS. The SRS is a 65 item assessment completed by the parent, teacher or caregiver of individuals aged 4–18 years yielding a quantitative measure of social ability. The areas assessed by the SRS include: social awareness, social information processing, capacity for reciprocal social communication, social anxiety/avoidance, and autistic preoccupations and traits. The total score reflects the degree of overall social impairment, and can distinguish children with ASD from other child psychiatric conditions.

### SNP genotyping and biomarkers

**Figure 1:**
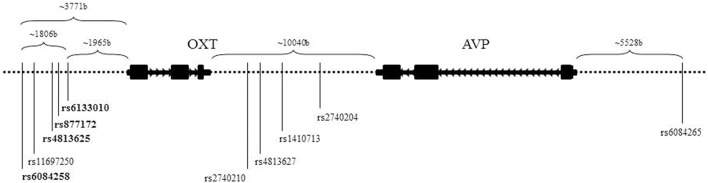
**Schematic of neurohormone genes, *OXT* and *AVP***. Located on chromosome 20, *OXT* and *AVP* are closely positioned and in opposite transcriptional orientations. The SNPs listed are the 10 genotyped SNPs utilized in this study. The bolded SNPs denote the SNPs used in the haplotype.

Tag SNPs were selected using data from the Caucasian CEU samples of HapMap (Haploview v.4, release 21; Barrett et al., 2005) and Tagger (de Bakker et al., 2005). The region spanned the adjacent genes *OXT* and *AVP*, and a ±20 kb flanking region. Other inclusion criteria were a minor allele frequency ≥0.10 and a pairwise *r*^2^ ≤ 0.80. rs6115776 in the most 5′ non-coding region could not be genotyped and has not been reported in other genetic association studies of OT-AVP genes. We successfully genotyped 10 SNPs across *OXT* and *AVP*. Figure 1 display the positions of the 10 genotyped SNPs and Supplementary Table 1 gives the linkage disequilibrium (LD) information for the four markers used in the haplotype. To genotype, DNA was extracted from 10 mL of blood using PureGene® DNA Purification Kit, then quantified with Quant-iT™PicoGreen® dsDNA Assay (Invitrogen, Carlbad, CA), and finally the samples were normalized to 10 ng/mL before genotyping.

Genotyping was performed, blinded to the affect status, family relationship, and demographic data utilizing TaqMan® SNP genotyping assays (Applied Biosystems, Foster City, CA). Standard TaqMan® SNP genotyping protocols were observed for PCR reactions. TaqMan® PCR reactions were done with 2.50 μL Universal Master Mix Amperase® UNG, 0.125 μL TaqMan® probe mix and 2.375 μL water for a 5 μL total volume. PCR conditions (Applied Biosystems™GeneAmp® PCR System 9700; Foster City, CA) were: oneAmpErase® step at 50.0°C for 2 min, one enzyme activation step at 95.0°C for 10 min, 40–99 alternating cycles of denaturation at 92.0°C for 15 s, and annealing and extension at 58.0°C for 1 min. Fluorescence intensity of the final PCR product was measured using a Roche Light Cycler Model 480-II (Hoffmann–La Roche AG, Basel, Switzerland, and Roche Light Cycler 480 Gene Scanning Software v.1.5, Basel, Switzerland).

In a subset of the sample, we were able to measure plasma levels of OT, allowing us to examine the relationships between pOT and the 10 tagged SNPs. From the 10 mL blood draw, 1 mL aliquots of plasma were stored at −80°C until analysis before assaying with radioimmunoassay (RIA) methods (Hammock et al., 2012). A relationship between OT and 5-HT has been observed in both animal models and ASD human studies (Hammock et al., 2012). To further explore this relationship, we examined relationships between the *OXT*-*AVP* tag SNPs and WB5HT levels. Whole-blood 5-HT was measured by high-performance liquid chromatography with fluorometric detection, as described previously (Anderson et al., 1981). Intra-assay and interassay coefficients of variation were 1.7 and 6.2%, respectively (Hammock et al., 2012). For WB5HT analysis, individuals taking medications that could potentially influence 5-HT as well as other psychotropic medications were excluded from analyses. In order to compare WB5HT values across age and race/ethnicity, z-scores were generated while adjusting for pubertal status and race/ethnicity. Pubertal status was determined based on their Tanner scale (Marshall and Tanner, 1969, 1970) or chronological age. Subjects were classified as pre-pubertal if their Tanner Scale was either I or II and post-pubertal if their Tanner Scale was greater than or equal to III. In the absence of a Tanner Scale (e.g., parents, young children, not examined), chronological age was used to create the puberty variable. Subjects with a chronological age less than 12 years were classified as pre-pubertal; whereas, subjects whose chronological age were greater than or equal to 12 years were classified as post-pubertal.

### Analysis

Before running the analyses, we checked for Mendelian errors and Hardy-Weinberg equilibrium. Utilizing PLINK v1.07 (Purcell et al., 2007) all SNPs in both samples were in Hardy-Weinberg equilibrium. Mendelian errors varied by SNP and were excluded from analysis on a per SNP basis. Utilizing FBAT, associations were examined between the tagged SNPs and ASD diagnosis, IQ measures, WB5HT (z-score), pOT, and other assessments measuring autism-related endophenotypes. Quantitative FBAT was carried out using FBAT v2.0.3 (Laird et al., 2000). Both dominant and additive models were tested. For each continuous phenotype of interest, associations between age and sex were explored, where significant, residuals after adjusting for these covariates were used as the adjusted phenotype in FBAT. If neither covariate was significant, residuals adjusted to the sample mean were computed and analyzed using FBAT. FBAT v2.0.3 was also used to test haplotype associations with ASD diagnosis and measured assessments. Haplotypes were created based upon significant associations in the 5′ region. Similar to the SNP analysis, Mendelian errors were excluded from analysis. After their findings, Hammock et al. (2012) suggested further examination of the relationship between WB5HT and pOT in a larger EA sample. We performed correlations and *t*-tests to examine not only the relationship between pOT and WB5HT, but also the relationship between these markers, age, and pubertal status. As in the Hammock et al. study, pOT was converted to fg/mL and log transformed to create a WB5HT-pOT ratio (WB5HT/log(pOT)). The distribution of log(pOT) and the ratio were tested utilizing SPSS v21 (IBM Corp Released 2012). Calculating the skewness z-score, both pre and post pubertal log(pOT) and the WB5HT-pOT samples were found to be normal with a z-score between −1.96 and 1.96. The lowest score was −1.13 (post log(pOT)) and the highest score was 0.52 (pre pOT). These results were confirmed by Shapiro-Wilk analysis (all *p*-values were greater than 0.07). After the analyses were conducted, we concluded that the biomarkers were normally distributed in both groups. An unpaired *t*-test was used to examine the relationship between the ratio and pubertal status.

### Expression quantitative trait loci (eQTLs)

We used ScanDB (Gamazon et al., 2010) to further investigate the functional impact of variants included in this study, which did include data from the expression levels of *OXT* and *AVP*. Specifically, we annotated genotyped variants (and SNPs in LD, *r*^2^ = 0.5, with genotyped variants) according to their association with expression levels in a previously published study of eQTLs in parietal and cerebellar brain regions (Chen et al., 2013). This approach and database were selected because of our previous research on enrichment of brain eQTL (Davis et al., 2012).

## Results

We examined associations between SNPs in *OXT* and *AVP* with ASD diagnosis and intermediate, quantitative phenotypes (Tables 2A,B). For the significant associations we found effect sizes were relatively small (φ_*c*_ < 0.10, *df* = 1—utilized for ASD diagnosis effect size; 0.016 < η^2^ < 0.129—utilized for remaining significant phenotypes) for all analyses displayed in Tables 2A,B. In the dominant model the most 5′ *OXT* tag SNP, rs6084258, was associated with ASD diagnosis (*p* ≤ 0.001). When restricting the analysis to the more ethnically homogeneous EA subsample the association remained significant for the A risk allele (*p* ≤ 0.006). Next, we investigated SNP associations with quantitative phenotypes of IQ (FSIQ, NVIQ, and VIQ), RRB, and social communication. The A-allele of rs6133010, near the 5′ region was found to be associated with lower FSIQ (*p* = 0.008), lower NVIQ (*p* = 0.010) and lower VIQ (*p* = 0.006), using the dominant model in FBAT. Similar to a previous ASD study (Yrigollen et al., 2008), rs2740204 was associated with RRB (dominant model: *p* = 0.036). There were two SNPs associated with social communication, rs4813625 for ABC–Social Withdrawal (*p* = 0.001) and rs1410713 for ABC-Inappropriate Speech (*p* = 0.021) in the dominant inheritance model. With Bonferroni correction (*p* ≤ 0.05/10 or 0.005) for 10 tag SNPs, only rs6084258 (in EA subsample for ASD diagnosis) and rs4813625 (in entire sample for social withdrawal) remained significant.

Table 2**Significant associations**.

**Table:** 

**Phenotype**		**SNP**	***p*-value**	**Inheritance Model**	**Z**	**Associated Allele**	**Risk Allele**
**(A)**							
Diagnosis	ASD	OXT rs6084258	0.001	Dominant	−3.30	G	A
IQ	FSIQ	OXT rs6133010	0.008	Dominant	−2.63	A	A
	NVIQ	OXT rs6133010	0.010	Dominant	−2.59	A	A
	VIQ	OXT rs6133010	0.006	Dominant	−2.78	A	A
Restricted/Repetitive Behaviors	ADOS—RRB	OXT-AVP rs2740204	0.036	Dominant	2.10	T	T
			0.022	Additive	2.30	T	T
Social Communications	ABC—Social Withdrawal	OXT rs4813625	0.001	Dominant	−3.25	C	G
			0.001	Additive	−3.41	C	G
	ABC—Inappropriate Speech	OXT-AVP rs1410713	0.021	Dominant	2.31	A	A
			0.036	Additive	2.09	A	A

**Table:** 

	**SNP**	***p*****-value**	**Inheritance Model**	**Z**	**Associated Allele**	**Risk Allele**
**(B)**						
WB5HT (z-score)	OXT rs4813625	0.027	Dominant	2.21	C	C
	OXT rs877172	0.033	Dominant	2.14	T	T
Plasma OT	OXT rs6084258	0.011	Dominant	−2.54	G	G
	OXT rs11697250	0.010	Dominant	−2.57	A	A
		0.039	Additive	−2.06	A	A
	OXT rs877172	0.002	Dominant	−3.17	G	G
		0.005	Additive	−2.82	G	G

The OXT SNPs are located in the 5′ region of OXT and the OXT-AVP SNPs are located in the 3′ region between the two genes. SNPs in these areas have been shown to be associated with ASD-related phenotypes in other studies. **(A)** summarizes the significant associations between tagged SNPs and ASD diagnosis and measured assessments in the entire sample. **(B)** displays the significant SNPs from the biomarker (WB5HTz, pOT) analyses in an EA subsample (WB5HT: N ≤ 108; pOT: N ≤ 38). Boxplots have been submitted as supplementary figures, to provide graphical detail about the associations between the significant SNPs and WB5HT (Supplementary Figures 1A–D) and pOT (Supplementary Figures 2A–F).

Given our significant ASD diagnosis association with rs6084258 and previously published associations with rs6133010 (Ebstein et al., 2009), we did haplotype analysis in the 5′ region (Figure 1, Table 2). The four-marker haplotype (rs6084258-rs4813625-rs877172-rs6133010) and all the permutations (two- and three-marker haplotypes) were analyzed in the whole sample (Table 3) and EA subsample (not shown). Haplotypes were significantly associated with IQ difference (VIQ-NVIQ), VIQ and social communication (ABC-Social Withdrawal and SRS Total). When restricting to an EA sample, ADOS-RRB was significant for rs877172G-rs6133010A (*p* = 0.035). These haplotype results need to be replicated in a larger sample.

**Table 3:** **Haplotype associations in the entire sample**.

**Phenotype**	**Markers**				**Haplotype**				***p*-value**	**Inheritance Model**	**Z**
IQ Difference	rs6084258	rs4813625	rs877172	rs6133010	G	C	G	A	0.040	Additive	2.06
	rs6084258	rs4813625	rs877172		G	C	G		0.040	Additive	2.06
		rs4813625	rs877172	rs6133010		C	G	A	0.040	Additive	2.06
VIQ		rs4813625	rs877172	rs6133010		C	T	A	0.045	Dominant	−2.00
ABC—Social Withdrawal	rs6084258	rs4813625	rs877172	rs6133010	G	G	G	A	0.015	Additive	2.43
	rs6084258	rs4813625	rs877172		G	G	G		0.015	Additive	2.43
					G	G	T		0.050	Dominant	1.96
	rs6084258	rs4813625		rs6133010	G	G		A	0.003	Additive	3.01
	rs6084258	rs4813625			G	G			0.002	Additive	3.08
	rs6084258			rs6133010	G			A	0.013	Additive	2.49
		rs4813625	rs877172	rs6133010		G	G	A	0.015	Additive	2.44
		rs4813625		rs6133010		G		A	0.001	Additive	3.38
						C		A	0.028	Additive	−2.20
SRS Total	rs6084258	rs4813625			G	C			0.046	Additive	−2.00

Haplotypes created from four SNPs in the 5′ region of OXT were analyzed for associations with ASD diagnosis, quantitative phenotypes, and biomarkers. Analysis was performed on the entire sample (shown) and an EA subsample (not shown). Each row lists a significant haplotype for each phenotype tested. Two specific combinations of markers, rs6084258-rs4813625-rs877172 and rs4813625-rs6133010, had more than one significant haplotype for ABC—Social Withdrawal.

Analysis of the biomarkers in a homogenous sample (EA: pOT N ≤ 54; WB5HT N ≤ 108) also yielded significant results (Table 2B). rs4813625C (*p* = 0.027) and rs877172T (*p* = 0.033) were found to be significantly associated with WB5HT with a dominant inheritance model. In addition to the WB5HT results, FBAT yielded significant associations (dominant model) with pOT levels and *OXT* SNPs rs6084258G (*p* = 0.011), rs11697250A (*p* = 0.010), and rs877172G (*p* = 0.002). Boxplots displaying biomarker level in relation to genotype of the significant SNPs are provided as Supplementary Figures (Supplementary Figures 1A–D, 2A–F). The correlations performed with the biomarkers and age yielded a significantly negative correlation (*r* = −0.261; *N* = 111; *p* = 0.003) between WB5HT and age. We also conducted analyses performed in 2012 by Hammock and colleagues in this larger sample and noted a significant relationship with WB5HT/log(pOT) and pubertal status [*t*_(31.92)_ = 3.51, *p* = 0.001]. The previous results used 11 years of age as an approximated pre-puberty cut-off whereas we used Tanner Scale less than III given that Tanner Scale III is assessed as mid-puberty in the literature.

Our final analyses looked at eQTLs to further investigate the functional impact of variants included in this study. According to eQTL annotations (Table 4), we found that genotyped variants (and those SNPs in LD with genotyped variants) show a low level of association with expression of nearby genes in parietal lobe and cerebellum, suggesting possible mechanisms for these variants. The best evidence for association with gene expression came from rs6076466 cis-association with RBCK1 (*p* = 5.467e-05), a ubiquitin-binding transcription factor. None of the variants were associated with expression of *OXT* or *AVP* in either brain region. These annotations should be interpreted with caution as differential expression across the brain can influence the power to detect SNP associations with expression levels. As more is known about OT/AVP expression in different brain regions, analyses should be expanded to compare regional differences. In addition, our analysis with ScanDB did not allow us to determine effect size for our finding; therefore follow up studies should not only extend expression and functional research but include effect size where possible.

**Table 4:** **Significant SNPs from eQTL analysis**.

**SNP**	**Gene**	***p*-value**	**cis/trans**
**CEREBELLUM**			
rs8184236	CDC25B	0.0043	cis
	C20orf96	0.0042	cis
rs2326055	NOP56	0.0086	cis
	SNORD57	0.0077	cis
	PANK2	0.0084	cis
rs11087565	RASSF2	0.0097	cis
rs6076466	TCF15	0.0062	cis
	RNF24	0.0052	cis
**PARIETAL**			
rs8184236	LRRN4	0.005855	cis
rs6076466	**RBCK1**	**5.467e-05**	**cis**
	GFRA4	0.0004464	cis
rs4815566	TMC2	0.003158	cis
	CRLS1	0.0007546	cis
rs2326055	RBCK1	0.004095	cis
	C20orf96	0.002497	cis

Genotyped variants displayed a low level of association with gene expression in the parietal lobe and cerebellum. The bolded SNP denotes the most significant association found in our analysis.

## Discussion

A relationship between the neurohypophysial hormones, OT and AVP, and their influence on social behaviors has been established across many mammalian species. The involvement of *OXT* and *AVP* in social behaviors has led investigators to research their possible dysfunction in disorders with social deficits as a major characteristic—including ASD and schizophrenia (Souza et al., 2010a,b; Teltsh et al., 2012). Results further support a connection between *OXT*-*AVP* and social behaviors, specifically a relationship with ASD, a disorder with significant social impairment. We investigated 10 tagged SNPs in *OXT* and *AVP*, revealing an association with ASD diagnosis and other ASD-related phenotypes such as IQ, RRB, and measures of sociality. Blood biomarkers, WB5HT and pOT, were also measured and analyzed, to determine mechanistic interactions with the SNPs and to explore their relationship to each other. We also combined SNPs to perform haplotype analysis picking four 5′ SNPs due to our findings with rs6084258 and previous findings with rs6133010. Additionally, we included eQTLs, to investigate expression in different brain areas.

Our approach in this study was to attempt to do deeper OT-AVP pathway phenotyping than previous studies. We predicted associations between *OXT*-*AVP* SNPs and phenotypes and blood biomarkers in an ASD sample. The main finding was a significant association with rs6084258 and ASD diagnosis. To date, there have been few associations with this SNP related to ASD or other phenotypes we researched. Another SNP of importance in this study was rs6133010, the most 3′ SNP of the analyzed haplotype. While previous research found rs6133010 to be associated with ASD diagnosis (Ebstein et al., 2009), our results showed a significant association with IQ (FSIQ, VIQ, and NVIQ) within our ASD sample. Another SNP of interest, rs2740204, has been associated with pharmacological treatment response, negative symptom improvement, and increased risk in schizophrenia (Souza et al., 2010a,b). Our results with this SNP (rs2740204) were similar to Yrigollen et al. (2008), who found an association with rs2740204 and RRB in an ASD population as measured by the ADI and ADI-R. We found an association with rs2740204 and RRBs as measured by ADOS in our sample. The SNP rs4813625, found to be associated with ABC-Social Withdrawal, has been implicated in both schizophrenia (Souza et al., 2010b) and reactions to stress in a healthy population (Love et al., 2012).

Reported haplotype results can vary due to the combination of markers analyzed or the variation within the study samples; therefore, these results need to be interpreted with caution and replicated across ethnically similar samples. The SNPs at either end of our haplotype consisting of 5′ *OXT* SNPs were of interest. As mentioned above rs6084258 and rs6133010 have been associated with diagnosis and different phenotypes as individual SNPs in ASD samples. In this haplotype analysis, we noted that when present, rs6084258G and rs6133010A were significantly associated with IQ and social communication measures (as assessed by ABC-CV and SRS).

With the relationship between OT and social behaviors, and the involvement of 5-HT in ASD and other disorders established, we performed additional analysis with pOT and WB5HT for possible associations with the tagged SNPs. Previous studies have also revealed a relationship between 5-HT and OT. Serotonin was shown to modulate OT release by interacting with different 5-HT receptors in the hypothalamus, an area where OT is primarily produced (Jørgensen et al., 2003). Lee et al. (2003) also observed increased pOT levels in healthy subjects when fenfluramine, a 5-HT agonist, was administered. In 2013, Dölen et al. explored the role of OT and 5-HT in social reward utilizing mice. They demonstrated that OT and 5-HT modify the mesocorticolimbic circuit by depressing excitatory synapses on to the nucleus accumbens, which is an important component of the mesocorticolimbic circuit reward system. This system has been implicated in rewarding behaviors that are key to survival, such as eating, drinking, and reproduction. Dölen et al. (2013) along with others, hypothesized that social interaction may be such a behavior and therefore utilizing a similar system of reward. Additionally, we have previously found a relationship between pOT and WB5HT in children and adolescents with ASD (Hammock et al., 2012). The findings above suggest interplay between OT and 5-HT.

Due to these findings we wanted to further explore possible interactions between the OT and 5-HT systems. Of note, the SNP found to be significantly associated with ASD diagnosis in this study (rs6084258) was also significantly associated with pOT. Although associated with pOT, it is with the opposite allele consistent with the negative correlation of the biomarker, decreased pOT in ASD, in this study. rs877172 was also found to be significantly associated with both blood biomarkers. rs877172 and rs4813625 (a SNP we found to be associated with both social withdrawal and WB5HT) were found to be associated with schizophrenia risk in the three-marker haplotype containing rs4813625, 877172, and 3761248 (C-G-C; Souza et al., 2010b). In order to assess the potential for a functional role of these variants in the regulation of OT and AVP, we explored a previously published eQTL analysis in both the cerebellar and parietal brain region tissues (Davis et al., 2012). The most significant association between a candidate SNP and gene expression was in parietal tissue with mRNA expression of RBCK1. RBCK1 is within a region of high linkage with ASD affection status and is located near *OXT* and *AVP* on chromosome 20 (Werling et al., 2014). Our results are consistent with limited mRNA expression of OT and AVP in the parietal and cerebellar regions of donor tissue samples in the Allen Brain Atlas (http://human.brain-map.org/). In these few adult, donor samples, OXT and AVP overexpression is highest within several hypothalamic regions. Future studies on neuropathology need to extend expression and functional research across developmental ages (fetal, early, pre puberty, post puberty, adult) and sexes.

As with many SNP variant studies of complex human disorders, there are some inconsistencies between our findings and the literature. We found rs2740204 to be associated with RRBs as measured by the ADOS, and rs6133010 to be associated with IQ scores. Ebstein et al. (2009) found that rs6133010 was associated with ASD diagnosis, and noted a haplotype association between rs6084265 and ASD diagnosis, IQ, and the Vineland. However, Hovey et al. (2014) found no associations with autistic-like traits (as measured ASD scores in the Autism-Tics, ADHD and other comorbidities inventory) and rs6133010 or rs2740204. Variable results could be due to many factors such as: heterogeneity of the ASD sample, diagnostic tool, age, sex, and ethnicity. By analyzing an EA subsample, we were able to control for inconsistencies due to ethnic diversity. In this subsample, rs6084258 remained significantly associated with ASD diagnosis, along with rs4813625 remaining associated with social withdrawal. Analysis of an EA subsample was also performed with the biomarkers (WB5HT and pOT) and haplotypes. Another factor could be the use of different assessments that may be identifying different aspects of similar behaviors.

Methodology could be a contributing factor to inconsistent findings in biomarker analysis, as discussed by McCullough et al. (2013) and Szeto et al. (2011). Over the past two decades investigators have reported higher pOT in ASD adults (Jansen et al., 2006), lower pOT in ASD individuals (Modahl et al., 1998; Andari et al., 2010; Yang et al., 2015), and no differences between ASD and non-ASD subjects (Miller et al., 2013) utilizing both enzyme immunoassay (EIA) and RIA. Taurines et al. (2014) found children with ADHD have lower pOT than age-matched typically developing or ASD children who had similar pOT levels. Limited, variable and changing antibodies, RIA vs. EIA, and differing collection and extraction methods influence outcomes along with differences between laboratory-generated RIA and commercially produced RIA and EIA kits. Variation in the age of the sampled population also affects results. In 1998, a study performed by Kumar and colleagues noted higher platelet 5-HT and its metabolite 5-HIAA in older vs. younger women (Kumar et al., 1998). Two points discussed by the authors in Hammock et al. (2012) were utilizing the Tanner scale or sex hormone status to further examine the relationship of 5-HT and pOT through development, and to replicate the study in a larger sample. In this study we addressed these points, finding a significant negative correlation with pubertal status (lower WB5HT post puberty) in a larger EA sample.

As with all studies, we had limitations to our research. Our ethnically heterogeneous sample is relatively small for genetic studies and our findings need to be replicated in larger samples. Given potential sex differences in the OT-AVP systems and in ASD (Carter and Jacob, 2012), future studies should include a larger female sample allowing researchers to explore these differences in detail. Future replications would also need to use a consistent OT plasma measuring methodology given the variability in the literature. There is a need in biomarker fields to determine a consistent protocol across studies, including extraction methodology and similar antibody sources until even more standardized proteomic methods are developed in lieu of immunoassays. Another limitation of our research is the preliminary nature of our eQTL analysis. A larger sample is needed for replication of our results, as well as, a deeper analysis comparing results from different databases, and possible differences in expression across age and between sexes.

The OT and AVP systems evolved in mammals to modulate various biobehavioral processes, including selective social bonding (e.g., pair, parent) and repetitive, territorial behaviors. Although, more is known about receptor distribution in animal brains, limited data is available about human brain pathways. Modulatory neurohormones like OT and 5-HT are likely to influence different neurocircuitry that affects social communication, RRBs, and learning/IQ. Therefore, it is possible that OT-AVP genes may influence multiple phenotypes. As dimensional approaches are taken with DSM-5 and the Research Domain Criteria (RDoC), further studies are needed to examine the multiple and overlapping circuitries of these and other phenotypes, such as the role of neuropeptide hormones in a range of disorders with social deficits. As seen in previous research there was overlap with SNP results in ASD and schizophrenia, although future studies will need to use the same measures of social and negative symptoms across disorders. Our study, along with others, have found pOT variation in ASD, social communication deficits in language, RRBs, ADHD, parental bonding, and trust in both patient and non-patient populations. Given that OT-AVP involvement could be related to phenotypes across disorders, other neurochemical systems are highly likely to be involved in these complex phenotypes. Future directions should examine involvement of interactive neurotransmitter systems. In 2013, Romero-Fernandez et al. found a rat dopamine-OT heteromer receptor (D_2_R-OTR; Romero-Fernandez et al., 2013), and Love et al. (2012) noted rs4813625 was found to modulate dopamine release in females under stress conditions. A 2015 study by Yang et al. examined the relationship between RRBs and the neurochemicals OT, 5-HT and cortisol (CORT). They found, in individuals with ASD, the levels of CORT, OT, and 5-HT were significantly associated with RBS-R total score. Within the domains of the assessment, CORT was positively associated with stereotyped and restricted behaviors, 5-HT with stereotypy and self-injurious behavior, and OT was negatively associated with compulsive, sameness and restricted behavior subscales. In addition, reverse translational animal studies may elucidate pathway mechanisms but note that results may differ across species (Bales et al., 2014). Our current study connects OT-AVP genes to ASD and several endophentypes. This intermediate phenotype approach will be important to examine the complex and intricate interplay between neurochemicals systems involved in neurodevelopmental disorders like ASD.

## Author contributions

Genotyping, analysis, and manuscript preparation were performed by SF, SJ. EK contributed to genetics data analysis throughout the study. ZY performed the oxytocin and precursor assay methods under the supervision of Dr. Marianna Morris. SG assisted with phenotype data collection and data management. EC contributed to sample collection and to manuscript preparation. SJ was the principal investigator for the study and coordinated the project. All authors read and approved of the final manuscript.

### Conflict of interest statement

The authors declare that the research was conducted in the absence of any commercial or financial relationships that could be construed as a potential conflict of interest.

## Abbreviations

EA: European Ancestry
pOT: Plasma Oxytocin
RRB(s): Restrictive/Repetitive Behavior(s)
SNP(s): Single Nucleotide Polymorphism(s)
WB5HT: Whole-blood Serotonin
WB5HTz: Whole-blood Serotonin z-score.
