# Supplementary material for: Variants in Adjacent Oxytocin/Vasopressin Gene Region and Associations with ASD Diagnosis and Other Autism Related Endophenotypes
Source: Front Neurosci. 2016 May 12;10:195. doi: 10.3389/fnins.2016.00195 (PMC4863894; doi:10.3389/fnins.2016.00195)
Supplement: Supplementary file 1 [file Table1.PDF]

Supplementary Material

Variants in adjacent oxytocin/vasopressin gene region and associations with ASD diagnosis and other autism related endophenotypes

Sunday M. Francis[1], Emily Kistner-Griffin[2], Zhongyu Yan[3], Stephen Guter[4], Edwin H. Cook[4], Suma Jacob[1]*

*** Corresponding Author:** sjacob@umn.edu

1 Supplementary Figures and Tables

1.1 Supplementary Table

	OXT rs6084258	OXT rs4813625	OXT rs877172	OXT rs6133010
**OXT rs6084258**		1.00 (0.68)	1.00 (0.30)	0.42 (0.02)
**OXT rs4813625**	1.00 (0.68)		1.00 (0.41)	0.86 (0.13)
**OXT rs877172**	1.00 (0.30)	1.00 (0.41)		0.74 (0.04)
**OXT rs6133010**	0.42 (0.02)	0.86 (0.13)	0.74 (0.04)	

Supplementary Table 1. Linkage disequilibrium table of the four marker haplotype. The haplotypes consisted of these four SNPs and the two- and three- marker permutations. The D’ and r^2 values (r^2 in parentheses) calculated using HapMap (https://hapmap.ncbi.nlm.nih.gov) are displayed in this table.
