# Supplementary material for: Variants in Adjacent Oxytocin/Vasopressin Gene Region and Associations with ASD Diagnosis and Other Autism Related Endophenotypes
Source: Front Neurosci. 2016 May 12;10:195. doi: 10.3389/fnins.2016.00195 (PMC4863894; doi:10.3389/fnins.2016.00195)
Supplement: Supplementary file 2 [file Image1.PDF]

Supplementary Material

Variants in adjacent oxytocin/vasopressin gene region and associations with ASD diagnosis and other autism related endophenotypes

Sunday M. Francis[1], Emily Kistner-Griffin[2], Zhongyu Yan[3], Stephen Guter[4], Edwin H. Cook[4], Suma Jacob[1]*

*** Corresponding Author:** sjacob@umn.edu

1. Supplementary Figures and Tables

1.2 Supplementary Figures

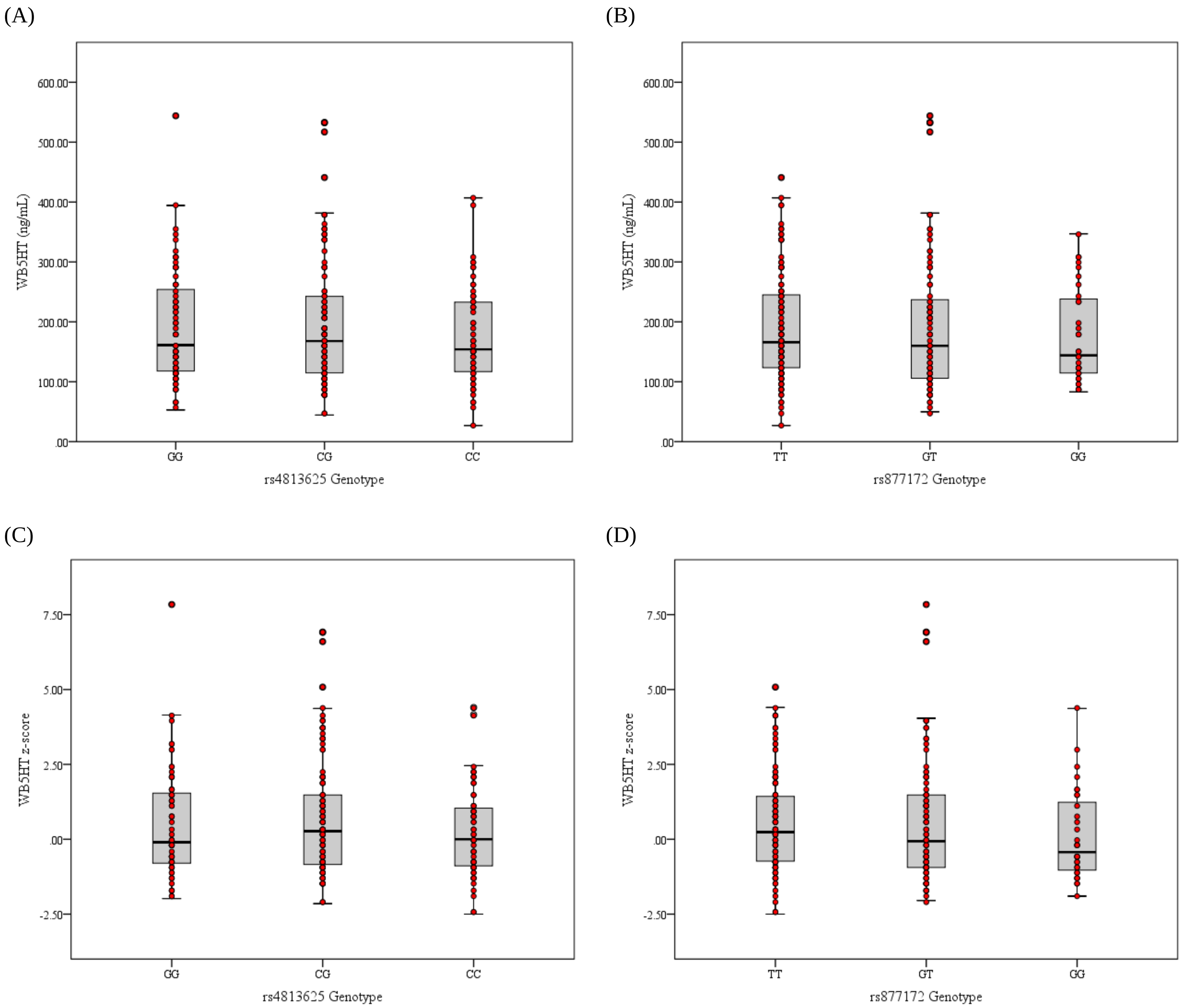

Supplementary Figure 1: Whole-blood serotonin levels by genotype. Figures 1A-B display WB5HT in relation to the genotype of the SNPs, rs4813625 and rs877172. Figures 1C-D display the relationship between WB5HT z-score (WB5HTz) and the genotypes of rs4813625 (p=0.033) and rs877172 (p=0.027). WB5HTz was utilized in the FBAT analysis.
