# Supplementary material for: Variants in Adjacent Oxytocin/Vasopressin Gene Region and Associations with ASD Diagnosis and Other Autism Related Endophenotypes
Source: Front Neurosci. 2016 May 12;10:195. doi: 10.3389/fnins.2016.00195 (PMC4863894; doi:10.3389/fnins.2016.00195)
Supplement: Supplementary file 3 [file Image2.PDF]

Supplementary Material

Variants in adjacent oxytocin/vasopressin gene region and associations with ASD diagnosis and other autism related endophenotypes

Sunday M. Francis[1], Emily Kistner-Griffin[2], Zhongyu Yan[3], Stephen Guter[4], Edwin H. Cook[4], Suma Jacob[1]*

*** Corresponding Author:** sjacob@umn.edu

1. Supplementary Figures and Tables

1.2 Supplementary Figures

(A)

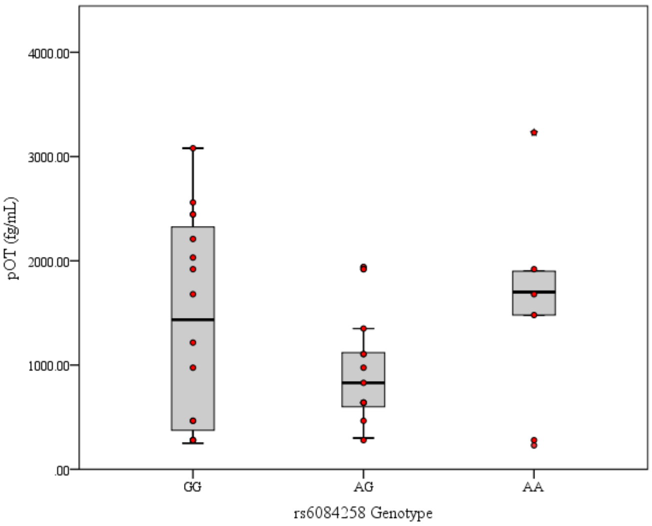
pOT (fg/mL)
4000.00
3000.00
2000.00
1000.00
.00
GG
AG
AA
rs6084258 Genotype

(B)

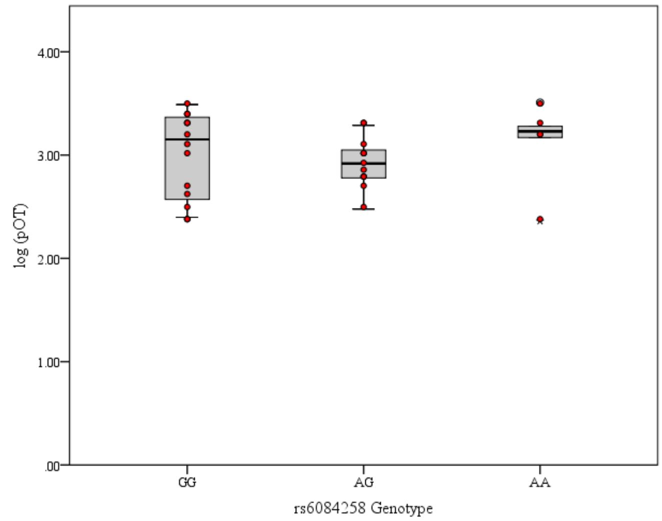
log (pOT)
4.00
3.00
2.00
1.00
.00
GG
AG
AA
rs6084258 Genotype

(C)

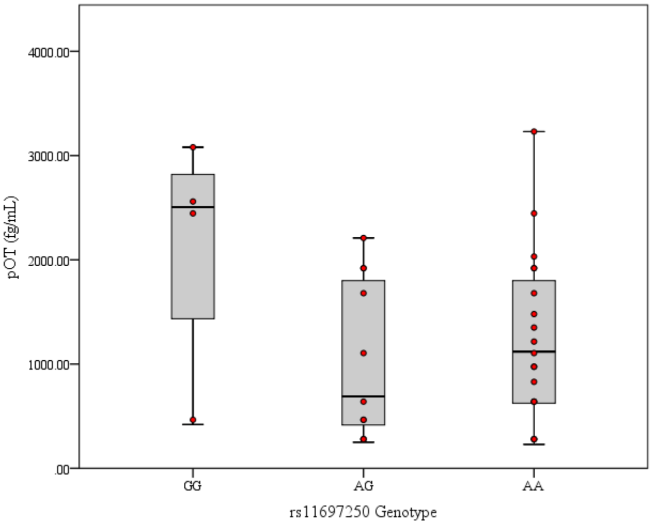
pOT (fg/mL)
4000.00
3000.00
2000.00
1000.00
.00
GG
AG
AA
rs11697250 Genotype

(D)

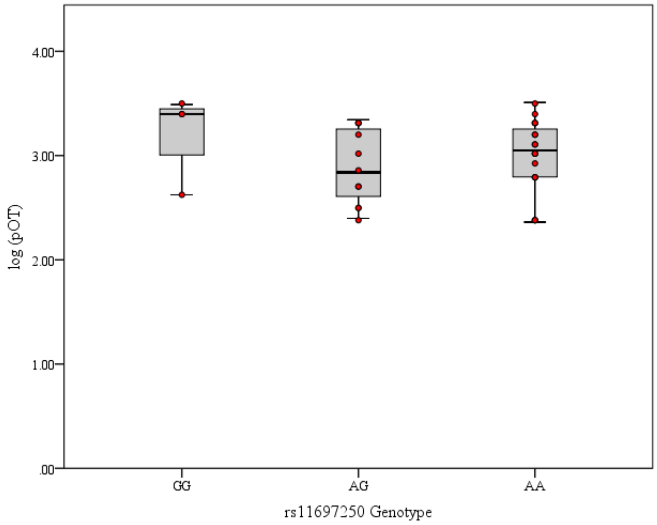
log (pOT)
4.00
3.00
2.00
1.00
.00
GG
AG
AA
rs11697250 Genotype

(E)

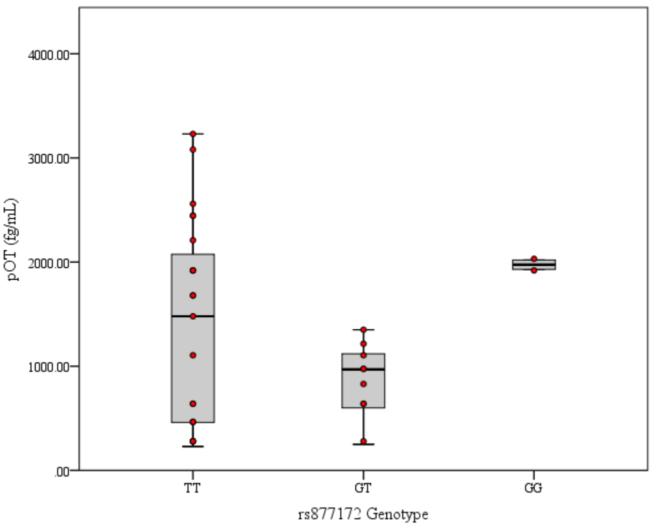
pOT (fg/mL)
4000.00
3000.00
2000.00
1000.00
.00
TT
GT
GG
rs877172 Genotype

(F)

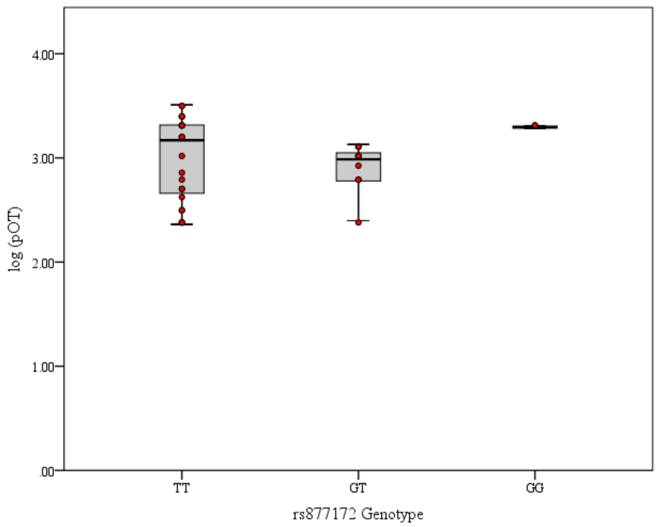
log (pOT)
4.00
3.00
2.00
1.00
.00
TT
GT
GG
rs877172 Genotype

Supplementary Figure 2: Plasma oxytocin levels by genotype. Figures 2A, C, and E display plasma oxytocin (pOT) in relation to the genotype of the significant SNPs, rs6084258 (p=0.011), 11697250 (p=0.010), and rs877172 (p=0.002). Plasma OT levels were used in the FBAT analysis. Figures 2B, D, and F display the relationship between log(pOT) and the genotypes of the SNPs listed above.
